# Sliding Wear Performance of AlCrN Coating on TiB_2_/Ti Composites at High Temperatures

**DOI:** 10.3390/ma14226771

**Published:** 2021-11-10

**Authors:** Remigiusz Michalczewski, Marek Kalbarczyk, Zbigniew Słomka, Edyta Osuch-Słomka, Maciej Łuszcz, Le Liu, Maksim Antonov, Irina Hussainova

**Affiliations:** 1 Łukasiewicz Research Network—Institute for Sustainable Technologies, ul. K. Pułaskiego 6/10, 26-600 Radom, Poland; marek.kalbarczyk@itee.lukasiewicz.gov.pl (M.K.); zbigniew.slomka@itee.lukasiewicz.gov.pl (Z.S.); edyta.slomka@itee.lukasiewicz.gov.pl (E.O.-S.); maciej.luszcz@itee.lukasiewicz.gov.pl (M.Ł.); 2 Faculty of Mechanical Engineering, Kazimierz Pułaski University of Technology and Humanities in Radom, ul. E. Stasieckiego 54, 26-612 Radom, Poland; 3 Department of Mechanical and Industrial Engineering, Tallinn University of Technology, Ehitajate 5, 19180 Tallinn, Estonia; le.liu@taltech.ee (L.L.); maksim.antonov@taltech.ee (M.A.); irina.hussainova@taltech.ee (I.H.)

**Keywords:** tribology, surface engineering, coating, TiB_2_/Ti composites, SPS, wear, adhesion, cermet

## Abstract

The aim of the study was to investigate effect of Ti/TiB_2_ composite composition and manufacturing technology parameters on the tribological behaviour of AlCrN coating-composite system. The AlCrN coating was deposited by PVD (Physical Vapour Deposition) method. The composites were manufactured by spark plasma sintering (SPS) from three variants of powders mixtures: Ti with TiB_2_, Ti6Al4V with TiB_2_ as well as Ti with B, using (five) different sintering temperatures. For each of the developed coating-composite systems, the wear resistance was evaluated using ball-on-disc SRV tester, at six temperatures (from room temperature up to 900 °C). The results confirmed that high-temperature wear resistance of the coating–substrate combination depends on Ti/TiB_2_ composite composition and manufacturing technology parameters. In the case of uncoated composite, two processes manage the wear at high temperatures: cracking propagation and surface oxidation. The presence of AlCrN coating on the composite surface protects the surface against deep cracking and surface oxidation. The composites of Group I, sintered at 1250 °C from a mixture of pure Ti and TiB_2_ (50/50 wt.% ratio) as well as Group III, sintered at 1350 °C from a mixture of pure Ti and B allow the achievement of a satisfactory surface quality, a high adhesion of the PVD coating and moderate wear at high temperatures. However, the composite made of pure Ti and B seems to be a better solution for temperatures exceeding 600 °C.

## 1. Introduction

In metal-working processes, the contact conditions between a workpiece and a tool are often extreme, with high contact pressures and high temperatures. The main damage mechanisms for tools, when processing difficult-to-machine workpiece materials, are adhesive material transfer, surface fatigue, and abrasive wear. New materials with excellent wear resistance at high working temperatures are demanded in modern manufacturing industry [1]. One of the most promising concepts relies on the design of high-temperature resistant, self-lubricating materials e.g., composites and multifunctional coatings [2,3]. One of the modern high-temperature resistant materials are TiB_2_/Ti ceramic-based composites prepared with the help of spark plasma sintering (SPS) [4,5]. Such composites are considered to be an alternative for tool steels because of their high ability to maintain mechanical properties at high temperature.

The composites which are the subject of present work, are based on titanium and titanium alloys and are widely used for many applications including metallurgy, aerospace, automotive industry, etc., due to a high specific strength, relatively low density, high-temperature stability and a good corrosion resistance [6]. Among the main limitations in using these alloys are insufficient hardness and low wear resistance. Furthermore, as a general rule the titanium alloys are not recommended for high-temperature application. The insufficient tribological performance of titanium alloys is mainly attributed to a non-protective tribo-oxide layer, which is generally thin, non-adherent, and brittle [7]. Therefore, the enrichment of the composites with a of refractory and hard phase, such as TiB_2_ and titanium, is considered to be a solution to improve mechanical properties and reduce wear rate [8]. Titanium diboride is one of the most suitable companions for Ti alloys providing an extremely high specific modulus, high hardness (around 30 GPa) and modulus of elasticity (about 565 GPa), and a relatively low coefficient of thermal expansion (7.2 × 10^−6^/K) [9].

To overcome the limitations such as insufficient tribological properties various approaches can be applied, including surface modification and hard coating deposition. Currently, there is no universal composition and structure of a coating that can be equally effective in a wide range of temperatures [10]. As an example, the increase of Al content in the coating can improve the oxidation resistance [11,12]. A certain progress has been observed in nanocomposite and multi-layered coatings such as in the case of VAlN or TiC/Al_2_O_3_/TiN [10,11,13].

One of the solutions might be a commonly used AlCrN hard coating which has very good high-temperature properties and a superior wear resistance as compared to often used TiN coating [14]. AlCrN coating is a common protective coating in the tool industry due to its high oxidation resistance and thermal stability [15] providing good tribological characteristics [16]. The properties of AlCrN coating are dependent on its chemical composition and structure [17].

To use the benefits provided by coatings and ceramic composites, it is crucial to consider their implementation at the design stage. It is well known that friction and wear characteristics are not intrinsic properties of materials [18], but a result of interactions between surfaces sliding against each other under certain test conditions, additionally highly influenced by an ambient temperature [19,20].

The tribological characteristics depends on many factors, such as material structure and surface finishing [21,22,23]. For high-temperature working conditions, the search for the most suitable TiB_2_/Ti composites technology and proper surface preparation, aimed on the PVD coating deposition, is still at an early stage of development. One of the main factors enabling the successful application of coatings is the ability to remain attached to the substrate under required operating conditions.

The previous study has shown that the TiB_2_/Ti composites preparation process is the factor which determines the PVD coating adhesion, which in practice can be evaluated only in the experimental manner [24]. The present study is a continuation of the previous work and shows the AlCrN PVD coating tribological performance, from the point of view of the impact of TiB_2_/Ti composite substrate composition and manufacturing technology parameters, with a special emphasis on coating wear mechanisms at high temperatures.

## 2. Materials and Methods

### 2.1. TiB_2_/Ti Composites

**Figure 1:**
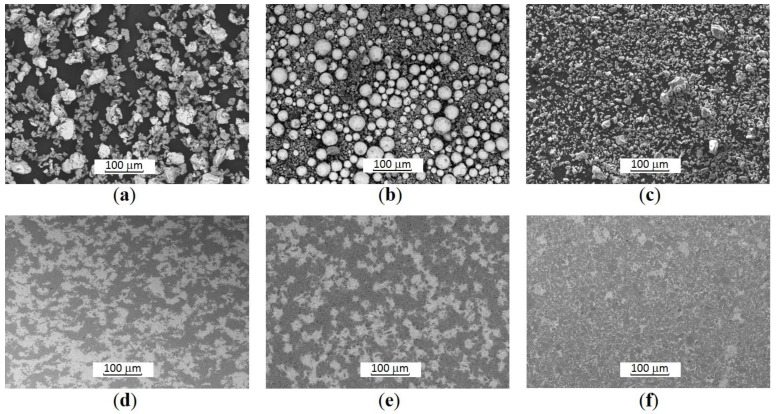
SEM images of the powders mixtures: (**a**) Group I (Ti-TiB_2_); (**b**) Group II (Ti6Al4V-TiB_2_); (**c**) Group III (Ti-B after SHS and subsequent disintegrator milling). SEM images of the SPS-ed samples: (**d**) Group I (at 50 MPa, 1250 °C, 15 min); (**e**) Group II (at 50 MPa, 1250 °C, 15 min); (**f**) Group III (at 50 MPa, 1350 °C, 15 min).

The ceramic-matrix composites with a high content of borides are assumed to be good candidates for high-temperature working applications by combining high hardness, good fracture toughness and possibility for self-lubrication [25]. Metallic phase of 15 wt.% provides a decrease in sintering temperature as well as an acceptable adhesion between the coating and a substrate [4]. Composites of Ti/TiB_2_ with a ceramic phase content of 85 wt.%, were prepared with the help of spark plasma sintering (SPS) of the pre-mixed powders of either Ti or Ti6Al4V alloy. To obtain the 15–85% weight ratio of metallic and ceramic components, the precursor powders of approximately 50–50% weight ratio for Ti or Ti6Al4V and TiB_2_ were mixed either by the conventional or disintegrator (DSL-175) system mixing; the specimens are designated as materials of Group I and Group II. The composites of Group III were fabricated from the powders synthesized by self-propagating high-temperature synthesis (SHS) of the elemental titanium and boron powders as detailed in [26,27]. Scanning electron microscope (SEM) (HRSEM Zeiss Merlin, ZEISS, Oberkochen, Germany) images of developed powders are shown in Figure 1 (the first row).

The composites were consolidated by SPS (FCT Systeme GmbH, Sonneberg, Germany) during 15 min at pressure of 50 MPa. A heating rate of 100 °C/min was applied to each sample despite the different sintering temperature ranged from 1050 °C for Ti6Al4V/TiB_2_ (materials of Group II) up to 1350 °C for Ti/TiB_2_ (Group I and III). The densified samples were of 9 mm thickness and 25 mm in diameter. The typical SEM images of the SPS produced composites of each group are displayed in Figure 1 (the second row).

**Figure 2:**
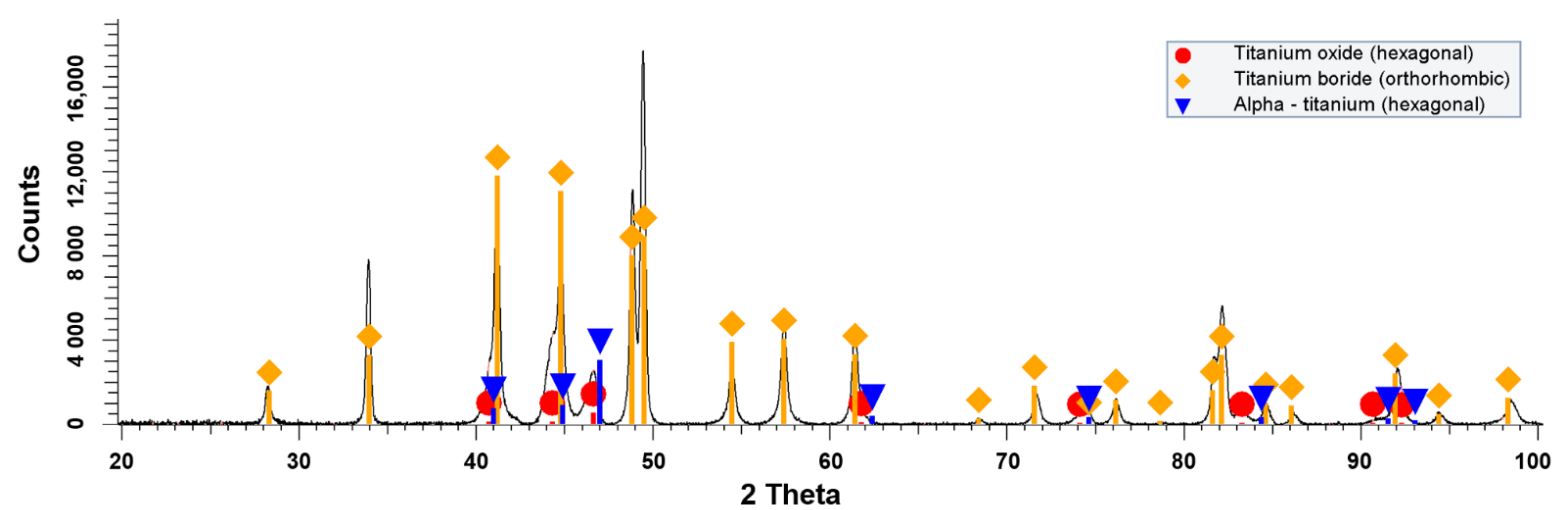
XRD pattern of the Group I specimen (Ti-TiB_2_, sintering parameters: 1250 °C, 50 MPa, 15 min).

XRD spectrum of the composite of Group I, measured using D8 Discover diffractometer, manufactured by Bruker (Bruker AXS GmbH, Karlsruhe, Germany), equipped with Co X-ray tube (Kα1 = 1.79026 [M2] Å) is presented in Figure 2. According to the obtained results, the XRD pattern contains titanium (hexagonal phase α-Ti), TiB (orthorhombic) and titanium oxide (hexagonal). The presence of TiB in the composite, is a result of the reaction between Ti and TiB_2_ via spark plasma sintering at 1250 °C.

After manufacturing, the samples were grinded and polished to obtain a sample surface without extreme irregularities. The details of surface finishing are given in [12].

The difference in particular phases mechanical properties and the porosity, has limited the ability to obtain a smooth surface to composite samples from Group I and III.

**Figure 3:**
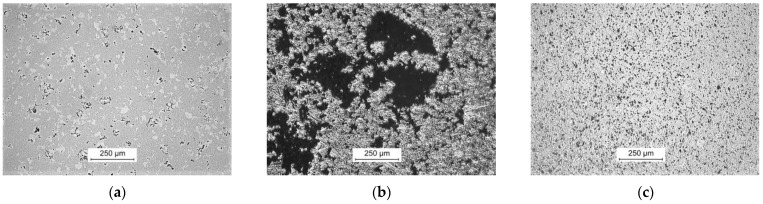
Optical images of the surfaces of composite samples after polishing process: (**a**) Group I (Ti-TiB_2_, 1250 °C); (**b**) Group II (Ti6Al4V-TiB_2_, 1050 °C); (**c**) Group III (Ti-B, 1350 °C).

The continuation of the polishing process leads to the exposure of new cavities. The porosity and phase differentiation of the finally obtained composite surfaces are presented in Figure 3.

The average roughness (Ra) measured by means of a non-contact 3D profiler (Taylor Hobson Ltd., Leicester, UK) are summarized in Table 1.

The surfaces of the composites from Group II have the 10-fold higher Ra values than the composite material of Group I and III. It is an effect caused by numerous pores of depth exceeding 30 µm.

### 2.2. AlCrN Coating

**Figure 4:**
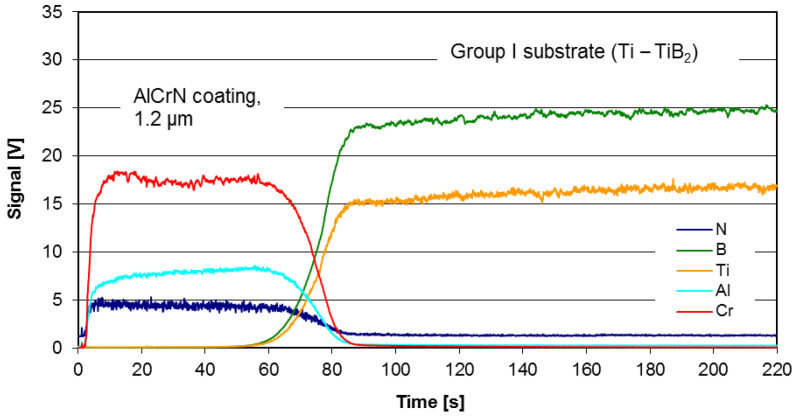
The GDOES profile: Group I substrate (Ti-TiB_2_) with AlCrN coating.

The discs made of various types of TiB_2_/Ti were coated using the commercial coating AlCrN-based (BALINIT^®®^ ALCRONA PRO made by Oerlikon Balzers, Polkowice, Poland) which exhibits excellent tribological characteristics at temperatures up to 1100 °C [28]. The deposition process parameters are not provided by the coating supplier. The chemical composition of the coating is 36.9 ± 0.2 at.% Al, 39.1 ± 0.2 at.% Cr and 21.2 ± 0.3 at.% N. The thickness and the hardness of the coating are respectively 1.2–1.3 µm and 3200 HV (0.05). The example of the depth profile of the AlCrN coating deposited on substrate of Group I (Ti-TiB_2_, sintering temp. 1250 °C), measured using JY 10000 RF Glow Discharge Optical Emission Spectrometer (GDOES) (Jobin-Yvon Horiba, Palaiseau, France), is presented in Figure 4.

The adhesion results of the AlCrN coating measured using the Rockwell C type intender with the angle of 120° and tip radius of 0.2 mm was used (REVETEST Scratch-Tester, manufactured by Anton Paar, (Anton Paar GmbH, Graz, Austria) are summarized in Table 2.

Three critical loads (LC1, LC2 and LC3) were determined for each coating. LC1 critical load denotes brittle tensile cracking, and it is considered to be a resistance to crack initiation. At LC2 load, first signs of chipping and delamination appear on the scratch. The LC3 critical load is reached when the coating is totally removed, and a substrate exposure takes place.

**Figure 5:**
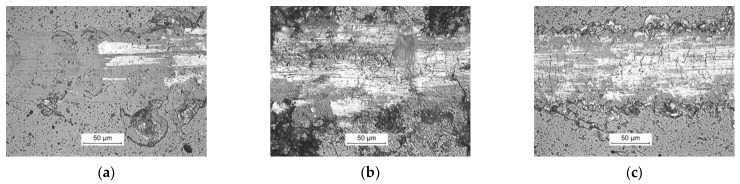
Optical images of the coating failure during the scratch test: (**a**) Group I substrate, sintering temp. 1250 °C, LC3 (76.4 N); (**b**) Group II substrate, sintering temp. 1050 °C, LC3 (22.8 N); (**c**) Group III substrate, sintering temp. 1350 °C, LC3 (69.5 N).

For the samples of group II (Ti6Al4V-TiB_2_), it turned out to be impossible to measure the coating adhesion—Figure 5.

The composites of Group II (Ti6Al4V-TiB_2_) require further preparation process optimization to obtain a less porous solid material, which would make it possible to improve the coating–substrate adhesion.

### 2.3. Wear Tests

**Figure 6:**
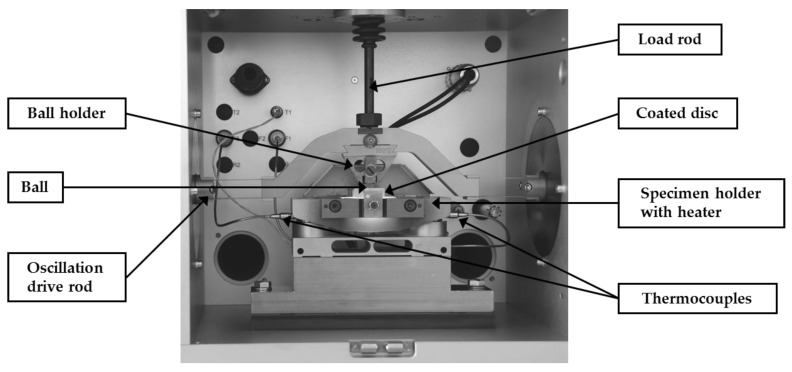
The oscillating ball-on-disc SRV tribosystem.

Sliding wear tests were conducted in ambient air using a high-temperature version of oscillating ball-on-disc SRV friction and wear tester (Optimol Instruments Prüftechnik, Munich, Germany). As a counterface, the Si_3_N_4_ ceramic balls (grade G20) of 10 mm in diameter were used. The test configuration is shown in Figure 6. The electromagnetic drive enables reciprocating movement between the upper ball specimen and the lower disc specimen. The normal load is applied by a servo motor and spring deflection mechanism.

The selected testing temperatures were RT (room temperature, 25 °C), 200, 400, 600, 750 and 900 °C using 1 °C/s increment and 1200 s temperature stabilization time. The following parameters were applied according to the previous research [29]: 5 N load, 1000 µm stroke, 10 Hz frequency, 300 s run time, and three test repetitions. The test conditions are set to obtain the wear high enough to be clearly measurable at wide range of test temperatures, while at the same time to avoid the complete coating removal in particular test run, which would be disqualifying for the test method.

The scars were investigated with the help of Talysurf CCI-Lite Non-contact 3D Profiler (Taylor Hobson Ltd., Leicester, UK) and the volumetric wear was measured by horizontal line approximation using TalyMap software.

The microstructure of the specimen’s surface was characterized by images recorded with the Hitachi (Tokyo, Japan) SU-70 Schottky emission scanning electron microscope (SEM) with a back-scattered electron (BSE) and secondary electron (SE) detection. The research was carried out in vacuum conditions (1 × 10^−8^ Pa) at an accelerating voltage of 15 kV.

## 3. Results and Discussion

Due to relatively low quality of Group II (Ti6Al4V-TiB_2_) samples, manifesting by approx. 8% high porosity, the wear resistance has been evaluated only for the samples from Group I (Ti-TiB_2_ with less than 2% of porosity) and Group III (Ti-B with around 2% of porosity). The materials of Group II require a further optimization to reach the level of homogeneity sufficient for the assessment of wear resistance.

The substrate composite of Group I, sintered at 1250 °C, was taken as a reference material for all conducted tests. The wear measurements were performed on specimens with the highest coating adhesion: Group I substrate (Ti-TiB_2_) sintered at 1250 °C with AlCrN coating, and Group III substrate (Ti-B) sintered at 1350 °C with AlCrN coating.

**Figure 7:**
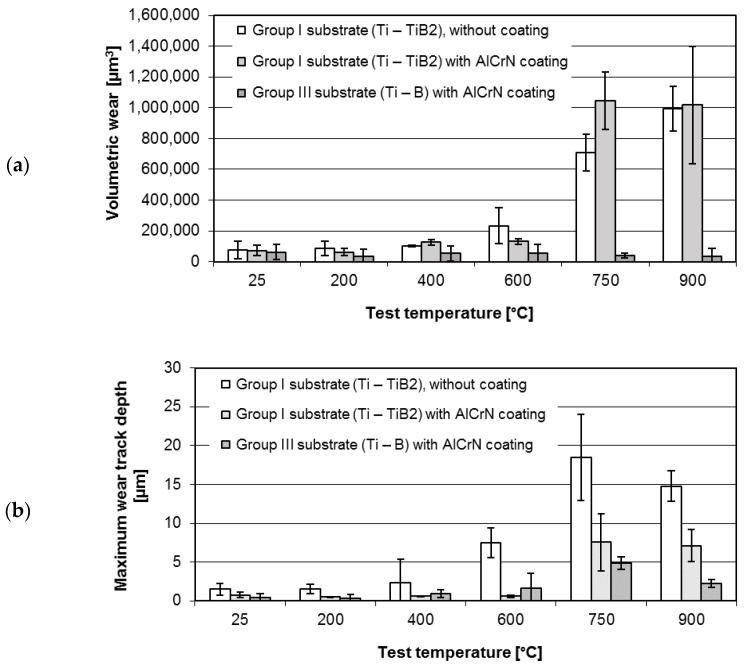
The average disc volumetric wear (**a**) and the maximum wear track depth (**b**).

The results of wear measurements for AlCrN coating deposited on two types of substrates, tested at various temperatures, are illustrated in Figure 7.

The volumetric wear (Figure 7a) and the maximum depth of the wear track (Figure 7b) show high dependence from the substrate type and test temperature. In most cases the temperature elevation increases the maximum wear track depth and the volumetric wear.

The volumetric wear of the uncoated composite is stable up to 400 °C, but further temperature rise leads to two-fold increase in volumetric wear at 600 °C and 7-fold and 10-fold at 750 °C and 900 °C, respectively.

**Figure 8:**
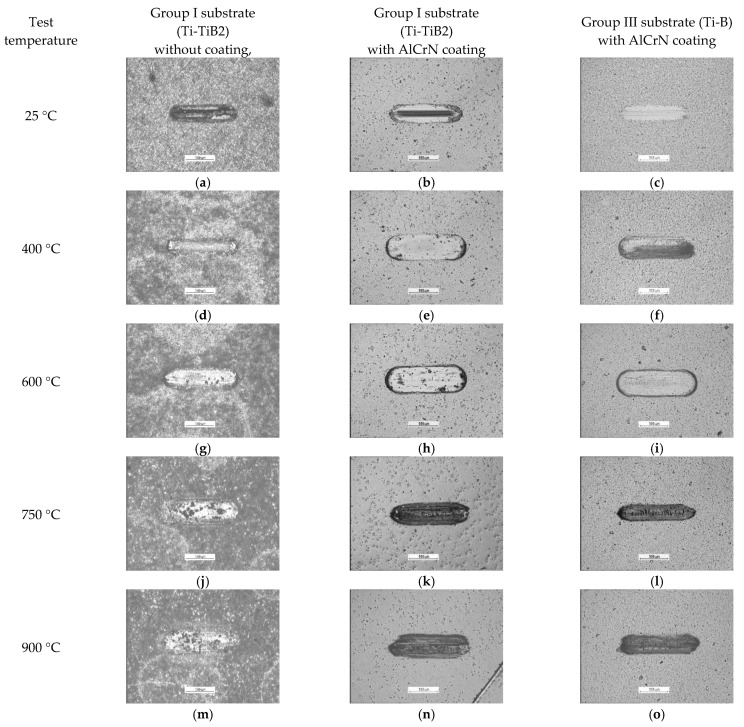
Evolution of the coating failure during the wear test (optical microscopy, scale bars show 500 µm for disc samples): (**a**) temp. 25 °C, Group I disc-uncoated; (**b**) temp. 25 °C, Group I disc with AlCrN; (**c**) temp. 25 °C, Group III disc with AlCrN; (**d**) temp. 400 °C, Group I disc-uncoated; (**e**) temp. 400 °C, Group I disc with AlCrN; (**f**) temp. 400 °C, Group III disc with AlCrN; (**g**) temp. 600 °C, Group I disc-uncoated; (**h**) temp. 600 °C, Group I disc with AlCrN; (**i**) temp. 600 °C, Group III disc with AlCrN; (**j**) temp. 750 °C, Group I disc-uncoated; (**k**) temp. 750 °C, Group I disc with AlCrN; (**l**) temp. 750 °C, Group III disc with AlCrN; (**m**) temp. 900 °C, Group I disc-uncoated; (**n**) temp. 900 °C, Group I disc with AlCrN; (**o**) temp. 900 °C, Group III disc with AlCrN.

In the temperature range from 25 to 600 °C (with 400 °C test exception), the wear resistance of the both coating-composite systems investigated in this study was improved, as compared with the resistance of the uncoated composite. At the highest test temperatures (750 °C and 900 °C) the volumetric wear of Ti-TiB_2_ composite without coating, was lower than for the coated one, while in the case of the maximum wear track depth the situation was opposite. Propagation of the coating failure during the wear test is presented in Figure 8.

The results presented in Figure 7 and Figure 8, indicate that the Ti/TiB_2_ composite composition has a significant influence on the tribological behaviour of AlCrN coating-composite systems. In the whole range of test temperatures, the volumetric wear of coated Group I substrate (Ti-TiB_2_) was higher than the wear of coated Group III substrate (Ti-B).

For uncoated systems at 600 °C, a certain number of large holes in the surface become visible, with an increasing tendency following the temperature growth (Figure 8). The presence of the coating changes the wear mode. The tests on coated specimens from RT up to 600 °C have resulted in the similar wear traces. At 600 °C, the short cracks on the wear traces on specimens from both groups can be observed, but their quantity for the Group III samples is larger. At 750 °C, the coating deposited onto material of Group I was totally removed from the surface, while the samples of Group III only demonstrated an intensification in damage.

**Figure 9:**
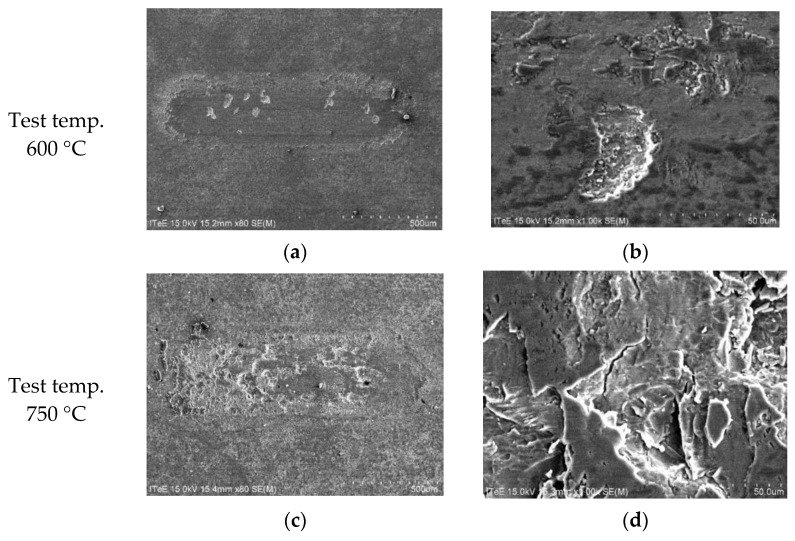
SEM micrographs presenting an overview of the contact zone on the disc made of Group I (Ti-TiB_2_) composite without coating, sintering temp. 1250 °C: (**a**) 600 °C; (**b**) a magnified view, 600 °C; (**c**) 750 °C; (**d**) a magnified view, 750 °C.

**Figure 10:**
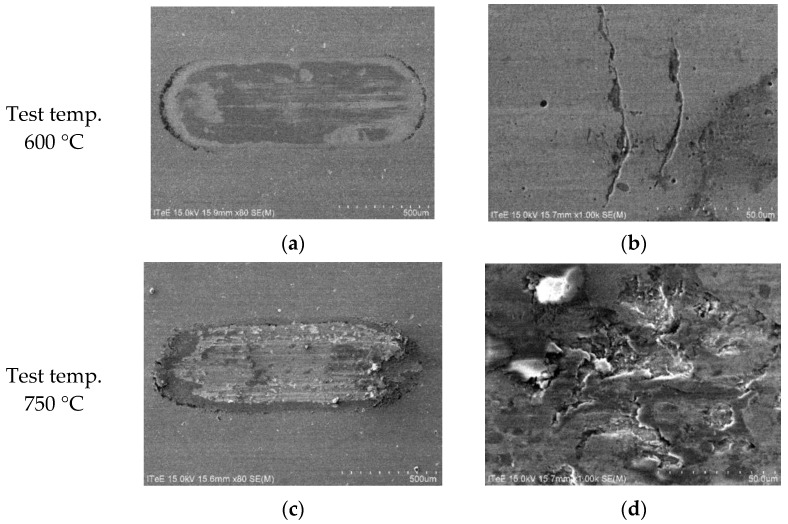
SEM micrographs presenting an overview of the contact zone on the disc made of Group I (Ti-TiB_2_) composite with AlCrN coating, sintering temp. 1250 °C: (**a**) 600 °C; (**b**) a magnified view, 600 °C; (**c**) 750 °C; (**d**) a magnified view, 750 °C.

**Figure 11:**
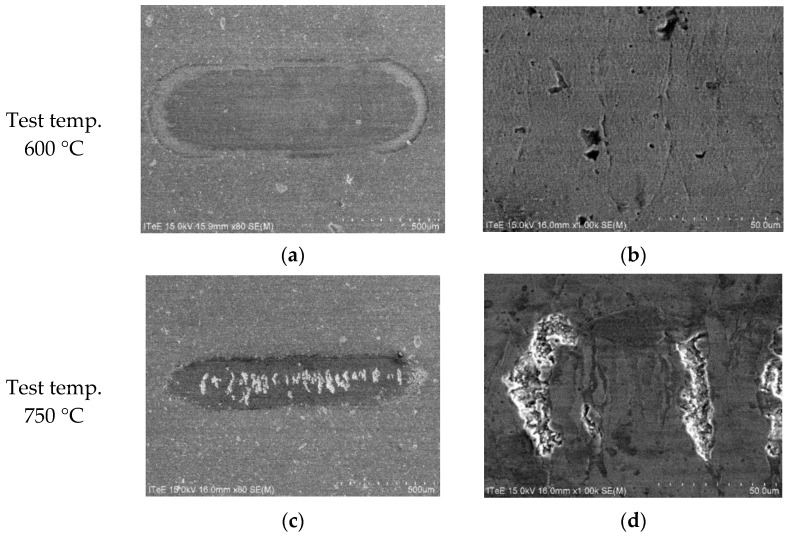
SEM micrographs presenting an overview of the contact zone on the disc made of Group III (Ti-B) composite with AlCrN coating, sintering temp. 1250 °C: (**a**) 600 °C; (**b**) a magnified view, 600 °C; (**c**) 750 °C; (**d**) a magnified view, 750 °C.

Further in-depth analyses were done to illustrate wear mechanisms in uncoated and coated systems, with a special focus on temperatures of 600 °C and 750 °C. The results of SEM observations are depicted in Figure 9, Figure 10 and Figure 11.

SEM micrographs taken from the wear track on the uncoated disc made of Group I (Ti-TiB_2_) composite revealed that the wear process at high temperatures is managed by the creation of the numerous cracks leading to the removal of large wear debris at 600 °C, and numerous cracks in the worn surface at 750 °C.

As shown in Figure 10a and Figure 11a, the AlCrN coating presented on the surface prevents against the crack propagation in the contact zone at 600 °C. For both disc materials only a certain number of cracks is visible on the surface, which is not followed by a substantial material removal (Figure 10b and Figure 11b). Such protection disappears at 750 °C with the coating removal. In the case of disc made representing Group I (Ti-TiB_2_) composite coated with AlCrN, the creation of numerous cracks on the wear track leads to a total material removal (Figure 10d). In the case of AlCrN coated Group III (Ti-B) composite the coating is removed only alongside the wear trace centerline resulting in (Figure 11d) transverse clusters of cracks and pits.

**Figure 12:**
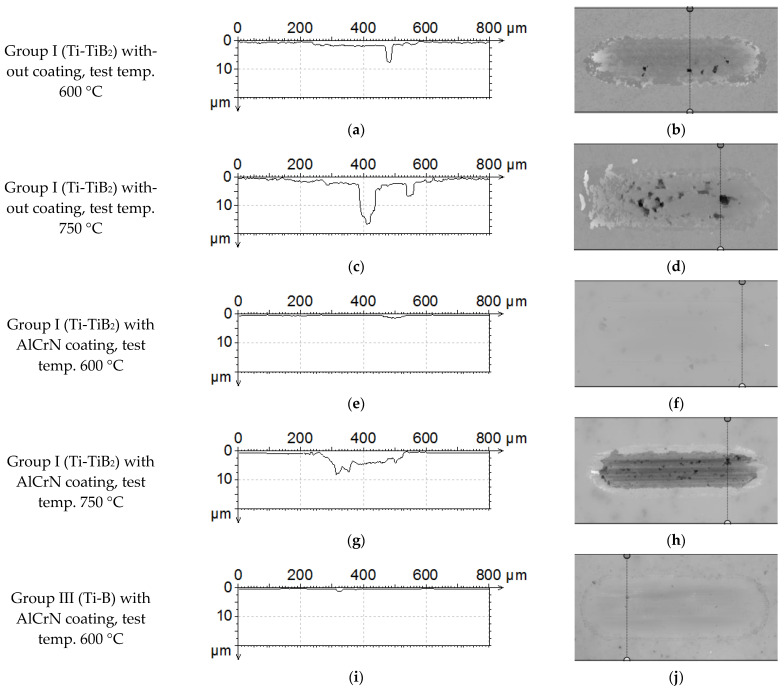

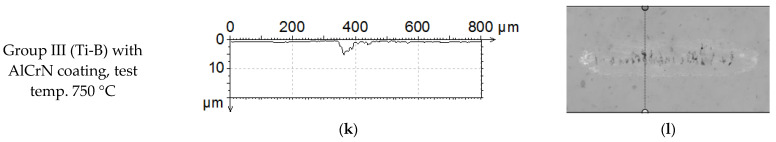
Wear trace profiles taken from the indicated positions: (**a**) Group I without coating, 600 °C, profile; (**b**) Group I without coating, 600 °C, position indicator; (**c**) Group I without coating, 750 °C, profile; (**d**) Group I without coating, 750 °C, position indicator; (**e**) AlCrN/Group I, 600 °C, profile; (**f**) AlCrN/Group I, 600 °C position indicator; (**g**) AlCrN/Group I, 750 °C, profile; (**h**) AlCrN/Group I, 750 °C position indicator; (**i**) AlCrN/Group III, 600 °C, profile; (**j**) AlCrN/Group III, 600 °C, position indicator; (**k**) AlCrN/Group III, 750 °C, profile; (**l**) AlCrN/Group III, 750 °C, position indicator.

The measurements of the crack depth were done using a non-contact profilometer. The cross-sections from the wear scars are presented in Figure 12. In the case of the uncoated composite surface, the material is removed creating numerous deep holes. The number and the depth of the holes depends on the test temperature. A higher temperature stimulates material removal even to almost 20 µm in depth (Figure 12c). At 600 °C, AlCrN coated samples from both groups provide similar and shallow wear scars with the average depth not exceeding 1.0 µm (Figure 12e,i). At 750 °C, in the case of sample from Group I, the coating is totally removed, resulting in a wide and deep wear scar (Figure 12g,h). In the case of samples from Group III, the extracted cross-sections indicate shallower wear scars but with deep cracks transverse to the motion direction (Figure 12k,l).

**Figure 13:**
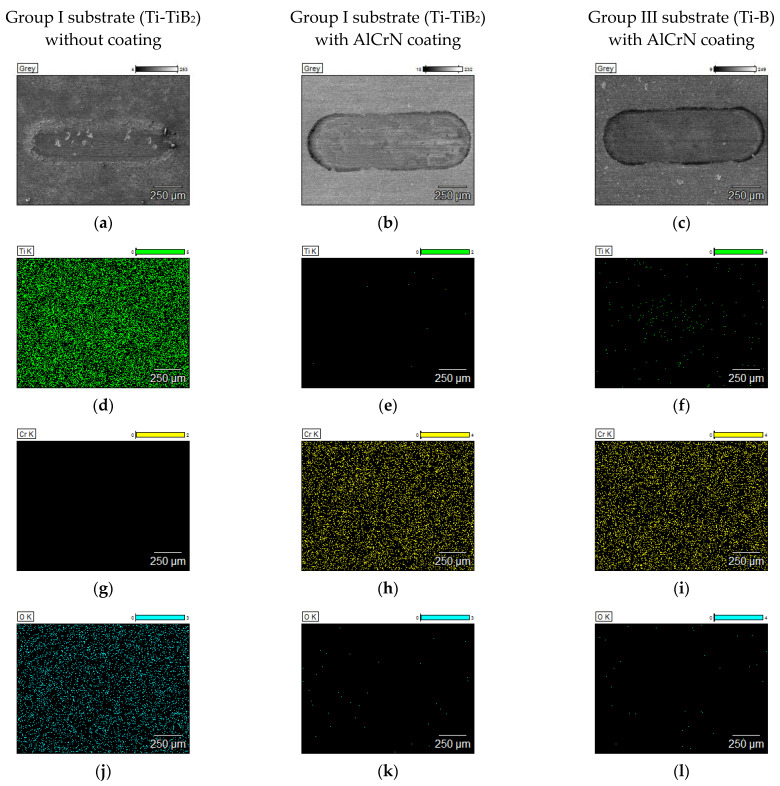
The EDS maps covering the wear tracks of the samples tested at 600 °C: (**a**) SEM image, Group I without coating, (**b**) SEM image, AlCrN/Group I, (**c**) SEM image, AlCrN/Group III, (**d**) Ti map, Group I without coating, (**e**) Ti map, AlCrN/Group I, (**f**) Ti map, AlCrN/Group III, (**g**) Cr map, Group I without coating, (**h**) Cr map, AlCrN/Group I, (**i**) Cr map, AlCrN/Group III, (**j**) O map, Group I without coating, (**k**) O map, AlCrN/Group I, (**l**) O map, AlCrN/Group III.

**Figure 14:**
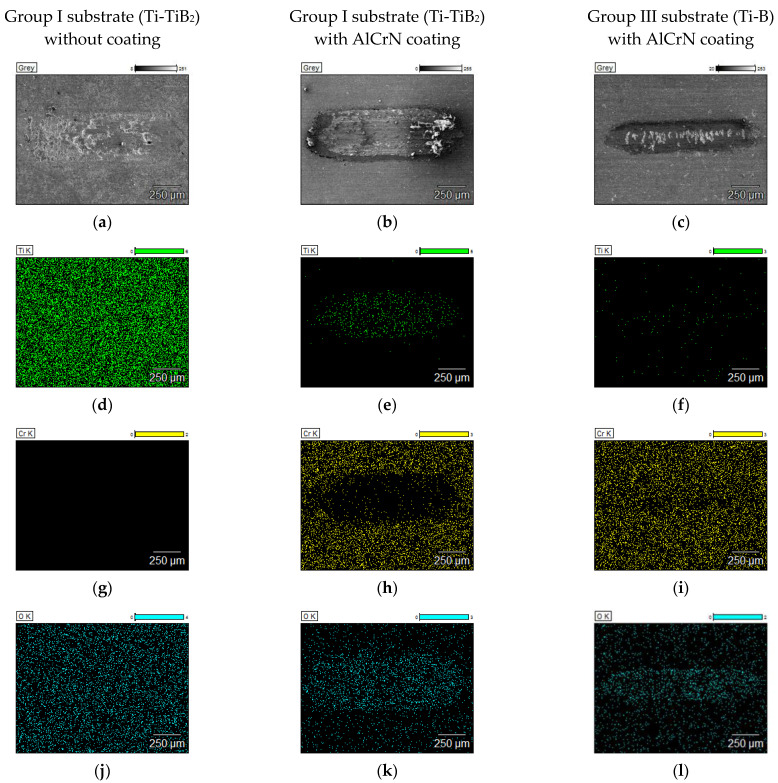
The EDS maps covering the wear tracks of the samples tested at 750 °C: (**a**) SEM image, Group I without coating, (**b**) SEM image, AlCrN/Group I, (**c**) SEM image, AlCrN/Group III, (**d**) Ti map, Group I without coating, (**e**) Ti map, AlCrN/Group I, (**f**) Ti map, AlCrN/Group III, (**g**) Cr map, Group I without coating, (**h**) Cr map, AlCrN/Group I, (**i**) Cr map, AlCrN/Group III, (**j**) O map, Group I without coating, (**k**) O map, AlCrN/Group I, (**l**) O map, AlCrN/Group III.

To verify the presence of the coating on the surfaces after tests at elevated temperatures, EDS maps from the were taken and are depicted in Figure 13 and Figure 14.

At 600 °C, AlCrN coated samples from both groups provide relatively shallow wear scars. For both disc materials only some cracks are visible in the surface which do not cause any significant removal of the coating material, what is confirmed by Cr maps given in Figure 13h,i). The coated surface is also free from oxygen. The uncoated composite is subjected to the oxidation even at temperature of 600 °C (Figure 13j). The AlCrN coating in effective way protects the composite against the oxidation. Only a few points with O detected are visible in the maps presented in Figure 13k,l. It is in agreement with the previous studies. Aluminium is commonly added to CrN systems in a range of 25–50% to improve the chemical stability via the formation of a protective Al_2_O_3_ layer preventing the diffusion of oxygen into the coating [30].

At 750 °C, for AlCrN coated samples from Group I substrate (Ti-TiB_2_), the total removal of the coating material is confirmed by the presence of Ti in the wear track (Figure 14e) and the lack of Cr (Figure 14h). For AlCrN coated samples from Group III (Ti-B) the coating is removed only in the areas of the deep cracks (Figure 14f,i). When the coating is removed, the uncovered composite material is subjected to the action of oxygen from the air.

At 750 °C, oxygen was detected in the case of uncoated composite, but also in the cases of AlCrN coated discs for which, after the tribological test, the coating interruption or removal was observed (Figure 14k,l).

## 4. Conclusions

The effect of Ti/TiB_2_ composite composition and manufacturing technology parameters on the tribological behaviour of AlCrN coating-composite system was comparatively investigated, with the focus on the composite-coating adhesion aspect and wear mechanisms differentiation at demanding high-temperature working conditions.

The following conclusions can be drawn from the study:

- The conducted research on ceramic-based discs of Ti/TiB_2_ has demonstrated that the selection of input powder mixtures and methods of their consolidation are the important issues to be taken into consideration for successful design of PVD coatings intended for high-temperature applications.
- The composites were manufactured by spark plasma sintering (SPS) from three variants of powders mixtures: Ti with TiB_2_, Ti6Al4V with TiB_2_ as well as Ti with B, using (five) different sintering temperatures. The composites made of Ti6Al4V-TiB_2_ require further optimization to reach the level of porosity and homogeneity adequate for proper finishing of the surface, which is crucial for coating deposition and the assessment of adhesion and wear resistance. The composites made of powders mixtures: Ti with TiB_2_, as well as Ti and B with different sintering temperatures are suitable for obtaining sufficient surface finish and to be coated in PVD process.
- The coating adhesion, measured using critical loads, obtained for the coating deposited on the surface of composites, depend on sintering temperature. The highest values of critical loads were obtained for the coated samples fabricated from the powder mixture prepared of the pure titanium and titanium diboride raw powders (Ti-TiB_2_). The first brittle cracks were found at loads a bit above 30 N, the partial delamination in the range of 36–40 N, and exposure of the substrate (total removal of the coating) at loads slightly above 75 N.
- The volumetric wear of the uncoated composites is found to be stable up to 400 °C, but further temperature rise, leads to two-fold increase in volumetric wear at 600 °C and 7-fold and 10-fold at 750 °C and 900 °C, respectively.
- The AlCrN coating can provide protection of the composites against the wear in the wide range of temperatures (from room temperature up to 900 °C).
- In the case of uncoated composite, two processes play a major role during wear: cracking propagation and surface oxidation. Both processes are temperature dependent. The presence of AlCrN coating on the composite surface protects the surface against deep cracking and surface oxidation.
- The composites of Group I, from a mixture of pure Ti and TiB_2_ as well as Group III from a mixture of pure Ti and B allow to the achievement of a satisfactory surface quality, high adhesion of the PVD coating and moderate wear at high temperatures. However, the composite made of pure Ti and B seems to be better solution for temperatures exceeding 600 °C.

## Figures and Tables

**Table 1:** The average roughness and hardness of the polished samples (before coating deposition).

Name	Temp.	Ra	HRC
	[°C]	[µm]	-
Group I (Ti-TiB_2_)	1250	0.091 ± 0.008	76 ± 2
	1450	0.067 ± 0.007	77 ± 1
Group II (Ti6Al4V-TiB_2_)	1050	1.599 ± 0.200	58 ± 9
	1150	0.853 ± 0.080	70 ± 2
	1250	0.461 ± 0.005	75 ± 2
Group III (Ti-B)	1050	0.034 ± 0.004	79 ± 1
	1150	0.036 ± 0.005	80 ± 1
	1350	0.044 ± 0.004	79 ± 1

**Table 2:** Scratch test results for AlCrN coating (progressive load from 0 to 100 N, incremental loading rate of 10 N/mm, 10 mm/min scratch speed, and 10 mm scratch length).

Name	Temp.	LC1	LC2	LC3
	[°C]	[N]	[N]	[N]
Group I (Ti-TiB_2_)	1250	28.0	38.5	77.8
	1450	36.9	38.9	75.4
Group II (Ti6Al4V-TiB_2_)	1050	not measurable	not measurable	not measurable
	1150	not measurable	not measurable	not measurable
	1250	not measurable	not measurable	not measurable
Group III (Ti-B)	1050	15.0	38.0	61.7
	1150	14.2	33.8	71.2
	1350	16.0	36.1	66.4

## Data Availability

The data presented in this study are available on request from the corresponding author.
